# Nanostructured Materials for Food Applications: Spectroscopy, Microscopy and Physical Properties

**DOI:** 10.3390/bioengineering6010026

**Published:** 2019-03-19

**Authors:** Shubham Sharma, Swarna Jaiswal, Brendan Duffy, Amit K. Jaiswal

**Affiliations:** 1 School of Food Science and Environmental Health, College of Sciences and Health, Technological University Dublin, City Campus, Cathal Brugha Street, Dublin D01 HV58, Ireland; shubh14sharma@gmail.com; 2 Centre for Research in Engineering and Surface Technology (CREST), FOCAS Institute, Technological University Dublin, City Campus, Kevin Street, Dublin D08 NF82, Ireland; swarna.jaiswal@outlook.com (S.J.); brendan.duffy@dit.ie (B.D.)

**Keywords:** nanotechnology, characterisation, microscopy, spectroscopy, food industry

## Abstract

Nanotechnology deals with matter of atomic or molecular scale. Other factors that define the character of a nanoparticle are its physical and chemical properties, such as surface area, surface charge, hydrophobicity of the surface, thermal stability of the nanoparticle and its antimicrobial activity. A nanoparticle is usually characterized by using microscopic and spectroscopic techniques. Microscopic techniques are used to characterise the size, shape and location of the nanoparticle by producing an image of the individual nanoparticle. Several techniques, such as scanning electron microscopy (SEM), transmission electron microscopy/high resolution transmission electron microscopy (TEM/HRTEM), atomic force microscopy (AFM) and scanning tunnelling microscopy (STM) have been developed to observe and characterise the surface and structural properties of nanostructured material. Spectroscopic techniques are used to study the interaction of a nanoparticle with electromagnetic radiations as the function of wavelength, such as Raman spectroscopy, UV–Visible spectroscopy, attenuated total reflectance Fourier-transform infrared spectroscopy (ATR-FTIR), dynamic light scattering spectroscopy (DLS), Zeta potential spectroscopy, X-ray photoelectron spectroscopy (XPS) and X-ray photon correlation spectroscopy. Nanostructured materials have a wide application in the food industry as nanofood, nano-encapsulated probiotics, edible nano-coatings and in active and smart packaging.

## 1. Introduction

Nanotechnology deals with the engineering of matter at atomic or molecular scale. Nano materials are novel and conventional materials engineered deliberately to be nanostructured and used frequently for many nanotechnology applications. The size range of the material in an unbound state, as an aggregate or as an agglomerate including external dimensions and/or internal structure for 50% or more of the particles in the number size distribution are measured in nanometres (nm), and the delimiting size range of the nanoscale and the microscales is 100 nm [1,2]. The major properties of a nano-system depend on the size of the matter. The interaction of nanoparticles (NPs) with the food environment is defined by the size of the NPs, agglomeration/aggregation state, composition, particle size distribution, shape (including length to diameter ratio for nanofibers and nanotubes), solubility, dispersibility, surface area, surface chemistry (including surface coating/functionalisation), surface charge density, surface hydrophobicity, water permeability, etc. In terms of properties and transport, nanoparticles are considered to be tiny entities that act as a complete unit. Other factors that define the characteristics of a nanoparticle are its physical and chemical properties, such as surface area, surface charge, hydrophobicity of the surface, thermal stability of the nanoparticle and its antimicrobial activity, and NPs are used in numerous food applications. Therefore, a legal framework had been setup to regularise some fundamental postulates for the safety and quiescence of the NPs in food. The properties that interest food technologists to include nanotechnology in their studies is the highly reactivity, adherence, improved bioavailability and bioactivity and surface effect of NPs.

Based on physical and chemical properties, the characterisation of a nanoparticle is carried out using different analytical techniques. It is necessary to characterise a nanoparticle in order to understand its structure and its interaction with the environment and to study its nanotoxicological properties and to gauge their safety when applying NPs to a food system. The influence of physical properties, such as size, shape, crystallinity, state of dispersion and the surface properties, effects health on exposure to the nanoparticle. A nanoparticle is usually characterised by using microscopic and spectroscopic techniques.

Microscopic technique is used to characterise the size, shape and location of NPs. The size distribution of NPs is expressed in mass and in number by producing an image of the individual nanoparticles. Elementary optical microscopy cannot be used to characterise a nanoparticle, as the size of a nanoparticle is below the diffraction limits of the visible light. Therefore, electron microscopy techniques and scanning probe microscopy are the prevailing methods. These microscopy methods sometime require the sample to be in the vacuum dried form or in probe tip geometry, which may cause the destruction of the sample. The microscopic methods mainly used to characterise nanoparticles are scanning electron microscopy, transmission electron microscopy (TEM), high-resolution transmission electron microscopy (HRTEM), atomic force microscopy (AFM) and scanning tunnelling microscopy (STM).

Spectroscopy is used to study the interaction of a nanoparticle with electromagnetic radiations as the function of wavelength. In some cases, it is also used to characterise the concentration, shape and size of nanostructured material. By using the principle of light scattering or the absorbance of light of different wavelengths, such as X-rays, laser light, ultraviolet light and visible light, a nanoparticle’s size, shape, concentration and composition can be determined. The spectroscopic techniques that are mainly used are Raman spectroscopy, UV—Vis spectroscopy, attenuated total reflectance Fourier-transform infrared spectroscopy (ATR-FTIR), dynamic light scattering spectroscopy (DLS), Zeta potential spectroscopy, X-ray photoelectron spectroscopy (XPS) and X-ray photon correlation spectroscopy.

Nanotechnology is of significant importance to the food industry as they provides the opportunity to create novel structure that enhances the texture, taste and the quality of food. The NPs (simple matrix, core shell, dispersion or combination) can be used in food industry in the form of nanotubes, nanoliposome, nanoemulsions, nanolaminate, nanocomposites, nanospheres, nanocapsules and nanofibers [3]. Nanomaterials incorporated into food could introduce or enhance the availability of principle nutrients, increase the shelf life, enhance the flavour and colour, develop new smart and intelligent packaging systems and improve safety and food traceability to monitor food during storage and transport.

## 2. Physical, Chemical and Biological Properties of Nanostructured Material

### 2.1. Particle Size and Surface Area

Particles of nanostructured material range from 1 to 100 nm in size. Nanostructured material (NSM) consists of interfacial layers, which have ions and organic and inorganic molecules that are responsible for all the alterations in the properties of the matter. NSM are mainly classified into four categories: zero-dimensional nanoparticles (0D NSM), one-dimensional nanoparticles (1D NSM), two-dimensional nanoparticles (2D NSM) and three-dimensional nanoparticles (3D NSM) [4].

Zero-dimensional nanomaterials are those that have sizes within the nanoscale range (no dimensions, or 0D, are smaller than 100 nm). These nanoparticles are amorphous, crystalline or polycrystalline. They are found in various forms and could be found either individually or agglomerated.

One-dimensional nanomaterials include nanorods, nanotubes and nanowires. These NPs could be amorphous, crystalline or polycrystalline in nature. 1D nanomaterials have a significant impact in nanodevices, nanoelectronics, nanocomposite materials and alternative energy resources.

Two-dimensional nanomaterials exhibit plate-like structures. Their chemical compositions vary and could be amorphous or crystalline in form. Also, they are either a single-layer or multilayer structure deposited on the surface of the substrate.

Three-dimensional nanomaterials are bulk materials that are not restrained to the nanoscale. Therefore, they are considered to have three random dimensions greater than 100 nm. With respect to their nanocrystalline structure, bulk nanomaterials can be formed by multiple arrangements of nanosized crystals. 3D nanomaterials contain bundles of nanowires or nanotubes and nanoparticle dispersion.

Primarily nanoparticles are characterised by evaluating the distributed particle size and morphology of the material. Electron microscopy helps in the evaluation of nanoparticle size, as well as morphology. Various studies have highlighted the effect of the size of nanoparticles on their application Nanomaterials can be used as bioactives in functional foods [5]. Reducing the particle size of bioactives may improve the availability, delivery properties and solubility of the bioactives and thus their biological activity, because the biological activity of a substance depends on its ability to be transferred across intestinal membranes into the blood [6]. Smaller particles are likely to combine during storage and help in the transportation of the dispersed nanoparticles. The smaller the size of the nanoparticles, the larger the surface area, which results in fast release. Polymer degradation is also affected by the size of the particles [7].

The specific surface area of a particle depends on its shape, size, pore size distribution, porosity and roughness. Specific surface area is used to design a heterogeneous catalyst with high specific surface area (e.g., silica, zeolites) [8]. The Brunauer–Emmett–Teller (BET) method is used to determine the specific surface area from nitrogen adsorption.

### 2.2. Surface Charge

One of the most important characteristics to determine various characteristics of a nanoparticle is the surface charge. It helps in the determination of the colloidal stability [9,10], nanostructure of the material, self-assembly of the nanoparticle [11], structure of nanocomposites, function of nanocomposites [12] and photocatalysis [13]. The electrostatic interaction of a nanoparticle with the environment, such as the bioactive compounds, is determined by the surface charge. The stability of colloidal nanomaterial is generally analysed by the zeta potential of its nanoparticles [14]. Zeta potential is the value of the surface charge of a nanoparticle at specific environmental conditions of the medium, such as pH, temperature, ionic strength, etc. It is measured by assessing the potential difference between the surface of shear and the outer Helmholtz plane and thus gives us the stability of the sample [14]. Zeta potential values are either positive or negative, ensuring the stability and avoiding the agglomeration of the particle. The pH of the solution determines the ionization of surface groups and, therefore, the final surface charge density [15]. Normally for the layer by layer (LBL) deposition technique, the pH of the solution should be selected so that the signs of the electrical charges on the particle surface and adsorbing polyelectrolyte are opposite, and the magnitude of the charges on both species is sufficiently high [16]. Many of the polyelectrolytes used in the food industry have ionisable groups that are relatively weak acids or bases.

### 2.3. Surface Hydrophobicity

Advancements in research have led to the development of various sophisticated tools for analysing a nanoparticle’s surface property. The property of a nanoparticle that affects the reaction of the nanoparticle in an aqueous environment and gives information regarding the transport of the nanoparticle is surface hydrophobicity [17]. The hydrophobicity of a nanoparticle influences the uptake and toxicity of the nanoparticle [17,18,19]; therefore, its characterisation helps in assessing the contact and hazards of the nanoparticle. The characterisation of hydrophobicity for a nanoparticle is not simple and has many challenges. Various techniques are utilised for determining surface hydrophobicity, such as biphasic partitioning, the measurement of the contact angle, hydrophobic interaction chromatography and the absorption of probes. Recent techniques, such as X-ray photon correlation spectroscopy, determine the hydrophobicity of the surface and identify the particular chemical groups on the surface of the nanoparticle [14]. Food emulsions are compositionally complex. Their droplets are stabilised to differing extents by proteins, small-molecule surfactants (emulsifiers) and, in certain cases, polysaccharides (hydrocolloids). To produce effective stabilisation of O/W emulsions, it has been found that the surface character of hydrophilic silica particles must be made somewhat hydrophobic.

### 2.4. Thermal Stability

The formation of the thermodynamic phase on the surface of the nanostructured material is due to the large volume of the interface fractions in the nanocrystalline materials, which differentiate between the granular and the nanocrystalline materials [20]. Several models have been applied to understand the metastable phase of formation in nanocrystalline materials.

In the bulk state, the thermodynamic condition for phase α to be stable over metastable phase β is Gα < Gβ [20]. In the case of nanocrystalline material, the contribution of interfacial energy term (Gint) to free energy cannot be neglected as in coarse-grained polycrystalline materials. The interfacial free energy of nanocrystalline materials can be expressed as:

∆Gint = 3 γint Vm/d

where Vm is the molar and d is the crystallite size. In the nanocrystalline state, β may become stable if Gα + Gα int > Gβ + Gβ int. In other words, if the rate of increase of total free energy (G + Gint) with the decrease in crystallite size of the α phase is higher than that of the β phase, the latter will become stable over the former below a critical crystallite size [20]. It has been found that the addition of nanoparticles enhances the thermal stability of the material. For instance, addition of nanoclay in polylactic acid (PLA) film enhances its thermal stability and mechanical properties [21]. Also, the thermal stability of a plant-derived bioactive compound, i.e., eugenol, was improved by encapsulation in chitosan-tripolyphosphate NP [22].

### 2.5. Antimicrobial Property

Nanoparticles that exhibits antimicrobial property either kill the microorganisms or decrease their growth rate. Antibacterial agents are broadly classified into two categories: bactericidal (kills the bacteria) or bacteriostatic (decreasing the growth rate of bacteria) [23]. The antimicrobial property of the nanoparticle depends on certain properties, such as size, surface area, morphology of the crystal reactive sites etc [23,24]. The main mechanism by which the nanoparticles exhibit antimicrobial property is not understood completely. By means of electrostatic interaction, nanoparticles can attach to the cell membrane and disrupt it. An important role is played by peptidoglycan on nanoparticles activity, but its effect also depends on various environmental conditions, such as pH, structure of nanoparticle, etc. The interaction of nanoparticles is also different for gram-positive and gram-negative bacteria. For gram-negative bacteria, the penetration of the particles depends on the size, charge and other properties of the material [25,26,27,28]. For instance, poly (butylene adipate-co-terephthalate)/silver nanoparticles (PBAT/AgNPs) composite films exhibited strong antimicrobial activity against both gram-positive (*L. monocytogenes*) and gram-negative (*E. coli)* food-borne pathogenic bacteria. The PBAT/AgNPs composite films with promising antimicrobial activity and improved film properties have a high potential for application as an active food packaging to secure food safety and to prolong the shelf-life of packaged foods.

## 3. Nanomaterial Characterisation by Microscopy

Nanostructures are very small in size and cannot be visualised by the naked eye, as well by optical microscope. Optical microscopes have the range for observing micron-structured material with a limited resolution. They are also unable to magnify more due to the divergence and limit of the light’s wavelength. Nevertheless, it is important to characterise the nanostructure structurally. Therefore, several techniques, such as scanning electron microscopy (SEM), transmission electron microscopy (TEM/HRTEM), atomic force microscopy (AFM) and scanning tunnelling microscopy (STM) have been developed to observe and characterise the surface and structural property of nanostructured material. All these techniques differ in their mechanisms but ultimately form a highly magnified image of the sample or the surface.

### 3.1. Scanning Electron Microscopy

**Figure 1:**
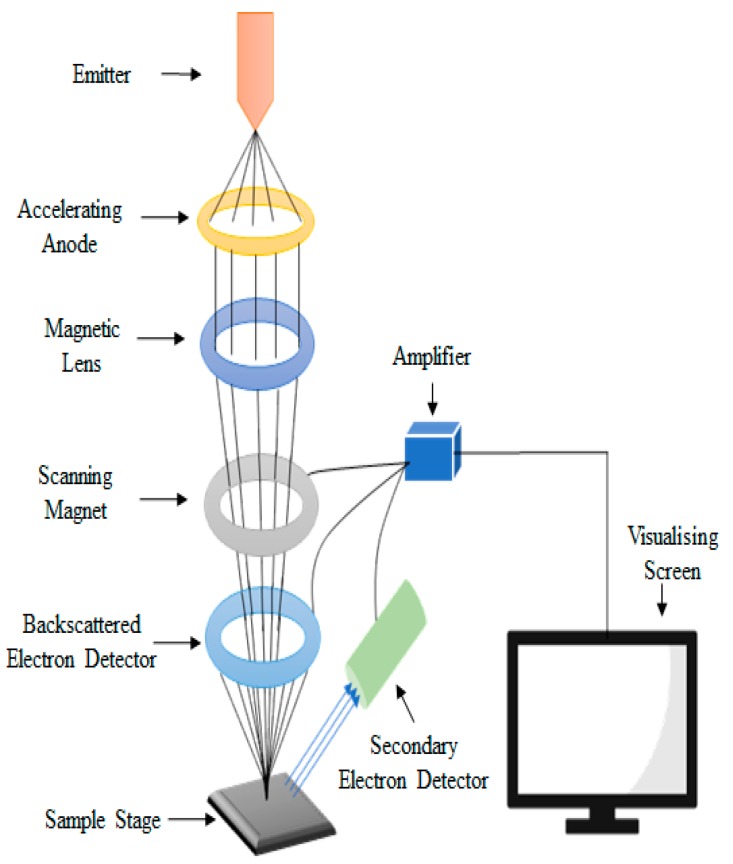
Scanning electron microscopy setup and its schematic diagram.

Scanning electron microscope (SEM) is a powerful electron microscope and a popular surface imaging technique. It images the surface of the sample by using electron high-energy beam scanning. SEM magnifies the image by using electrons rather than the light used by conventional microscopes. Sometimes, there is no clear differentiation between the nanoparticles of the sample, predominantly when the nanoparticles are inclined to agglomerate. The resolution of the image by SEM depends on the interaction of the specimen with the electron beam (Figure 1) [29].

The interaction of atoms when a beam of incident electron strikes the surface of the sample lead to the formation of secondary electrons, back-scattered electrons, diffracted electrons, and specific X-rays, which considerably depend on the surface morphology, topography and chemical constituent [30]. Secondary electron imaging SEM forms a very high-resolution image and can capture details of size up to 5 nm. SEM also uses specific X-rays and back-scattered electrons from the sample to form an image and to detect the elemental structure of the sample [31]. The high-resolution abilities of SEM make it suitable for finding the nanoscale features of the nanostructured material, which are significant to their properties and application.

In classical SEM, a beam of monochromatic electrons, induced by the emitter, are condensed and accelerated by the accelerating anode [29]. These streaming electrons are then deflected to the magnetic lens, which make them thinner and more coherent [32]. Further, these beams are passed through the scanning coil, which scan in a grid approach over the surface of the sample. The surface of the sample must be conductive for the beam of the electron to study the surface of the sample by SEM. The interaction of the sample and the electron beam generate secondary electrons, back-scattered electrons, x-rays, visible rays, absorbed electrons and diffracted electron. These numerous electrons containing information about the sample are detected and visualised on a screen. Secondary electrons are more sensitive for the lighter elements then the back-scattered electrons. Zhou et al. [33] studied the particle size of a whey protein film incorporated with TiO_2_. The samples of the film were vacuum dried at room temperature, placed on a metal stub and then gold was spat for the conductivity. Images were taken at 4500× magnification.

De Britto et al. [15] studied the morphology and particle size of *N*,*N*,*N*-trimethyl chitosan nanoparticles as a vitamin carrier system by using SEM. They casted nanoparticles on the microscope slide, fixed in the specimen stub and then used a very thin gold coating to avoid charging under the electron beam. The SEM images revealed the smooth and spherical morphology of the nanoparticles with regular distribution over the surface of the glass. Biddeci et al. [34] demonstrated a biopolymer film formed by pectin matrix with modified halloysite nanotubes containing essential peppermint oil and possessing thermosensitive antimicrobial properties. The SEM images in their study indicated that the halloysite tubular shape is conserved in the functionalised halloysite nanotube.

SEM gives a highly magnified three-dimensional image with a very high resolution. SEM is used to image the surface structure of the nanocomposite polymer, nanoparticles, nanofibers and nanocoating on the surfaces [29]. SEM also gives information regarding the purity of the sample. Moreover, it gives the degree of aggregation and determines the position of the secondary and tertiary nanostructure. SEM gives us the degree of dispersion of the nanoparticles on the sample. SEM application could be limited as the electron beam can damage the polymer of the nanoparticles. They also frequently require information about the sizing distribution [14]. Moreover, they are costly and time consuming.

### 3.2. Transmission Electron Microscopy (TEM) and High Resolution Transmission Electron Microscopy (HRTEM)

Transmission electron microscopy is a high-resolution structural and chemical characterisation microscopy technique [35]. An electron beam is transmitted through an ultra-thin sample and interacts while passing through it. The advancement in transmission electron microscopy facilitated the direct imaging of the structure of the atom of the solid and the surface by gathering information about the size, crystallinity, shape and the interaction of the particle. TEM creates a very high-resolution image of around 0.1 nm. It is also an important technique for chemical characterization as the beam of the electron passing through a very small diameter (less than 3 nm) of a nanocrystal [29].

A beam of electron emitted by the emitter can pass from the two condensers, which make the beam thin, small and coherent. Just before the interaction of the electron beam with the sample, all the high angle electrons are removed by the condenser aperture. The interaction of the beam of the electron leads to the determination of some scattered and unscattered electrons [30] which are detected by the detector. An important precaution to be taken into consideration when performing TEM measurements on nanoparticles containing samples is that they can be susceptible to the highly energetic electron beam of the TEM instrument. Beam susceptibility makes it very difficult sometimes to carry out electron diffraction studies on certain nanoparticles.

The preparation of a sample for TEM is a complex process. The sample must be very thin as the electrons are directly transmitted through the sample; therefore, the sample size is generally less than 150 nm. If a very high resolution is required, then it must be even below 30 nm. Chen and Wagner [16] converted their vitamin E emulsion nanoparticle sample into powder. Then, using the spraying process, the average particle size was reached around 5 nm or less, and a sample was then dissolved in water. The images were then taken using cryogenic transmission electron microscopy.

### 3.3. Atomic Force Microscopy (AFM)

The atomic force microscope (AFM) is a scanning probe microscope, which is designed to measure local properties. The local properties are friction, magnetism and height. Scanning probe microscopy (SPM) scans over the small area of the sample, measuring the local properties. It is a very-high-resolution type of SPM, with a demonstrated resolution in the order of fractions of a nanometre, more than 1000 times better than the optical diffraction limit [36].

AFM has three strengths: force measurement, imaging and manipulation. In force measurement, the measurement of the forces between the sample and the probe is conducted by AFM as a function of their mutual separation. This can be applied to perform force spectroscopy to measure the mechanical properties of the sample, such as the sample’s Young’s modulus, a measure of stiffness.

For imaging, the probe reaction towards the force applied by the sample is used to form the topography (three-dimensional shape) at a very high resolution of the sample surface. This could be attained by raster scanning the positions of the sample with respect to the height and the tip of the probe that accords with constant probe sample interactions.

In manipulation, the properties of the sample can be changed or controlled by applying force between the tip and the sample [37]. Some examples of manipulation are atomic, scanning probe lithography and local stimulation of cells.

AFM can also be used to measure other properties, such as an image, often with similarly high resolution. Examples of such properties are mechanical properties, such as stiffness or adhesion strength, and electrical properties, such as conductivity or surface potential. In fact, most SPM techniques are extensions of AFM that use this modality.

To study biological structures through AFM, they must be well attached to a smooth solid substrate. These substrates provide them with a support to resist the lateral forces applied by the scanning tip. The substrates that are mostly used are mica, silicon oxide and glass. Zhou et al. [33] studied titanium dioxide (TiO_2_)/whey protein isolate (WPI) film by AFM imaging functioning in tapping mode. They used micro-fabricated silicon cantilever tips with 299 kHz of resonance frequency and 20–80 N/m of spring constant.

De Moura et al. [38] obtained AFM images of silver nanoparticle in a cellulose-based antimicrobial food packaging in contact mode using a silicon nitrate tip with 0.06 N/m of spring constant

### 3.4. Scanning Tunnelling Microscope

Scanning tunnelling microscope (STM) is an instrument for imaging surfaces at the atomic level. It was invented by Gerd Binning and Heinrich Rohrer in 1981. The good resolution for STM is at 0.1 nm lateral resolution and 0.01 nm depth resolution [39]. It can be used in vacuum, air, water, gas and various other liquids at temperatures ranging from near zero kelvin to over 1000 °C. The whole concept is based on quantum tunnelling.

The components of an STM include a scanning tip, piezoelectric-controlled height, x, y scanner, coarse sample-to-tip control, vibration isolation system and computer. When a conducting tip is brought very near to the surface to be examined, a bias (voltage difference) applied between the two can allow electrons to tunnel through the vacuum between them [29,39]. The output (tunnelling current) is a function of applied voltage, tip position and the local density of states (LDOS) of the sample. Information can be obtained by analysing the current as the tip’s position scans across the surface and is usually displayed image form. STM can require extremely clean and stable surfaces, sharp tips, excellent vibration control and sophisticated electronics.

The resolution of an image is limited by the radius of the curvature of the scanning tip of the STM. Additionally, image artefacts can occur if the tip has two tips at the end rather than a single atom; this leads to “double-tip imaging”—a situation in which both tips contribute to the tunnelling [29]. Therefore, it is essential to develop processes for consistently obtaining sharp, usable tips.

Curri et al. [40] used STM to study a novel nanoparticle system for biosensor application. They immobilised CdS quantum dots on gold and recorded the sample in air at a voltage of 0.5 V and a reference current of 1 nA. From the STM images, they found that the NPs maintain their original size without being agglomerated in the immobilised state.

## 4. Nanomaterials Characterisation by Spectroscopy

### 4.1. Raman Spectroscopy

Raman spectroscopy is based on the phenomenon of scattering of light. In this technique, high intensity light is scattered with a probability that one in million photons will group with the rotational and the vibrational sample state and emit slightly diverse wavelength of light. Raman spectra are of very low intensity. The whole process depends upon inelastic scattering of laser light (monochromatic) known as Raman scattering. The interaction of the laser with phonons or molecular vibrations and other excitations result in a shift in the energy of the photons [41]. The shifts could be bidirectional (upward and downward). The change in the shift gives information about the phonon modes in the specific system. The effect comes into existence when the laser light interacts with the electron cloud of molecules [42]. The Raman effect occurs when light impinges upon a molecule, interacts with the electron cloud of the bonds of that molecule and an incident photon excites one of the electrons into a virtual state.

Stokes Raman scattering is generated when a molecule gets excited from the ground state to a virtual energy state and then relaxes into a vibrational excited state [29]. Anti-stokes Raman scattering is defined when a molecule is already in an elevated vibrational energy state and reaches to its ground state after scattering [41]. In order to exhibit the Raman effect, the amount of deformation of the electron cloud or the molecular changes in polarizability is required with respect to the vibrational coordinate [42]. The change in the intensity of the Raman scattering can be determined by the change in the amount of polarizability, and the vibrational level is equal to the Raman shift.

Raman spectroscopy helps to provide the fingerprint of a molecule, which can help in the identification of the molecule in the range of 500–2000 cm^−1^. Many practical applications are based on Raman gas analyser. Raman spectroscopy is used to characterise materials, measure temperature and find the crystallographic orientation of a sample.

Raman active fibres, such as aramid and carbon, have vibrational modes that show a shift in Raman frequency with applied stress. Polypropylene fibres also exhibit similar shifts. The radial breathing mode is a commonly used technique to evaluate the diameter of carbon nanotubes [29].

Surface enhanced Raman spectroscopy (SERS) is a fast and very sensitive analytical method and is used to detect or quantify trace amounts of pesticides in raw vegetables and fruits [43]. Fan et al. [44] showed that the feasibility of applying SERS for detection must be at least 1 mg/g for phosmet and 0.5 mg/g for carbaryl in apples. Luo et al. [45] analysed thiabendazole and phosmet, which has a broad range of applications in the analysis of other trace amounts of chemicals contaminants in food.

### 4.2. Ultraviolet–Visible Spectroscopy

Ultraviolet–visible spectroscopy refers to absorption or reflectance spectroscopy in the UV–visible spectral region. Absorption spectroscopy refers to a technique that measures the absorption of radiation with respect to the function of the wavelength or frequency. The absorption affects the identify colour in the visible range [29,46]. The atoms and molecules undergo transitions that are evident in the electromagnetic spectrum. The molecules, which hold n-electrons and π-electrons can absorb ultraviolet or visible light in the form of energy to excite these electrons into higher orbitals. The electron excitation tendency is directly proportional to the capacity to absorb the wavelength of the light.

UV–Vis spectroscopy is often used in chemistry for quantitative analytics, such as highly conjugated organic compounds, biological macromolecules and transition metal ions. Spectroscopic analysis is observed mainly in solutions, but it is not limited in other states of matter. The solutions of transition metal change colour when the electrons excite from one state to another after absorbing light.

Souza et al. [47] studied quercetin-loaded lecithin/chitosan nanoparticles as a functional food. The UV–Vis absorption spectra of free quercetin and quercetin NPs were obtained at the spectral range of 200–700 nm at 1.0 nm intervals. M. Esmaili et al. [48] studied UV-visible spectra of curcumin in the presence of various concentrations of Beta casein-micelle as a nano vehicle.

### 4.3. Attenuated Total Reflectance Fourier-Transform Infrared Spectroscopy (ATR-FTIR)

This process of spectroscopy, which includes the infrared light, falls into the prism in such a way that the angle exceeds the critical angle for internal reflection. This produces an evanescent wave at the surface (reflecting). The change of the evanescent wave is measured, producing a spectrum, which is then subject to a Fourier transform. This technique is a very reliable and efficient fingerprinting method [49]. This technique is used to characterise and identify many substances. The main strength of this techniques is its IR radiation, which is an analytical technique to obtain spectra from different states of matter. This has been used to analyse solids, liquids and gases by means of transmitting the infrared radiation directly through the sample. The sample is in a liquid or solid form, and the intensity of the spectral features is determined by the thickness of the sample. Typically, this sample thickness cannot be more than a few tens of microns [49]. This helps in the determination of the absorption or the transmittance of the IR radiation through the sample.

The technique of attenuated total reflectance (ATR) has, in recent years, revolutionised solid and liquid sample analysis, because it combats the most challenging aspects of infrared analysis, namely sample preparation and spectral reproducibility. ATR is an IR sampling technique that provides excellent quality data and the best possible reproducibility of any IR sampling technique. It has revolutionised IR solid and liquid sampling by the following means:

- Faster sampling;
- Improving sample-to-sample reproducibility;
- Minimising spectral variance.

Shankar et al. [50] obtained FTIR spectra of silver nanocomposite films with polylactic acid and lignin. The spectra were recorded as 32 scans per sample at a resolution of 4 cm^−1^. Shankar et al. [51] studied FTIR spectra to characterise chitin nanofibril-reinforced carrageenan nanocomposite films. Chitin and Chitin nano fibrils (CNF) were obtained using an ATR-FTIR spectrophotometer in the range of 4000–500 cm^−1^.

### 4.4. Dynamic Light Scattering Spectroscopy (DLS)

Dynamic light scattering (DLS) is a technique that can be used to analyse the size distribution profile of small particles in any solution. The fluctuations can be easily determined and analysed by the magnitude of the intensity or by the function of photon autocorrelation. With respect to the time domain analysis, the autocorrelation function generally decays starting from zero-delay time, and faster dynamics due to smaller particles lead to faster decorrelation of scattered intensity trace [52]. It is also used to investigate the behaviour of complex fluids, such as concentrated polymer solutions.

In DLS, a beam of monochromatic laser light is directed through a biomolecule solution and fluctuations in scattered light intensity are analysed. The experiment is non-invasive, requires only 12 μL of sample and can, in a matter of minutes, provide information about the size and homogeneity of biomolecules.

Sample characteristics, such as aggregation, folding or conformation can be monitored as a function of various preparations, solvent conditions, temperature or time. The system is used to pre-screen samples under the conditions in which the calorimetry experiment is planned. The instrument can be operated between 4 and 60 °C. It is equipped with software that outputs information about the hydrodynamic radius (in the sample size range of 0.5–1 μm) along with the percentage distribution of various components in the sample. Small traces of aggregation or oligomerisation can be checked and corrected for by improving the buffer conditions or choosing the optimal temperature for the calorimetry experiment [53].

A monochromatic light source, usually a laser, is shot through a polariser and into a sample. The scattered light then goes through a second polariser where it is collected by a photomultiplier, and the resulting image is projected onto a screen.

All the molecules in the solution are being hit with light, and all the molecules diffract light in all directions. The diffracted light from all the molecules can either interfere constructively (light regions) or destructively (dark regions) [52]. This process is repeated at short time intervals, and the resulting set of speckle patterns is analysed by an autocorrelator that compares the intensity of light at each spot over time. DLS delivers a quick and appropriate evaluation of the size of nanoemulsions. Therefore, it is used to evaluate the size distribution and the stability of nanoemulsions [54].

### 4.5. Zeta Potential Spectroscopy

The Zeta potential is also referred as “electrokinetic potential”. Many nanoparticles or colloidal particles have a surface charge when they are in suspension. When an electric field is applied, the particles move due to the interaction between the charged particle and applied field. The direction and velocity of the motion is a function of particle charge, the suspending medium and the electric field strength. Particle velocity is then measured by observing the Doppler shift in the scattered light. The particle velocity is proportional to the electrical potential of the particle at the shear plane, which is zeta potential. Thus, this optical measurement of the particle motion under an applied field can be used to the determine zeta potential.

Particle motion under an applied electric field is known as electrophoresis. The sample particles are suspended in a solvent at known refractive index n, velocity η and dielectric constant ε. The sample is irradiated with laser light of wavelength λ [53]. An electric field with strength E is applied. Due to the electric field, the particles are moving. Because the particles are moving, the scattered light at angle ϑ is measured, and the particle velocity V is determined from the frequency shift. Mobility is then readily obtained as the ratio of velocity to the electric field strength V/E. Zeta potential is found using the most common model known as the Smulochowski model.

$$U=\frac{\left(\lambda .V.d\right)}{2.E.n.\sin\left(\frac{\theta }{2}\right)}$$

The electric potential of a surface is the amount of work that needs to be done to bring a unit’s positive charge from infinity to the surface without any acceleration. The inner layer consists predominantly of ions/molecules with opposite charge to that of the particle (Stern layer). Beyond the Stern layer, the electrostatic effects due to the surface charge on the particles decreases as per Debye’s law, which states that with the distance of each Debye length, the field decreases by a factor of 1/e [53]. Although, mathematically, this electrostatic effect extends until infinity; experimentally, it is only present until a few nm from the particle surface [55]. Due to the electrostatic field of the charged NPs, a diffuse layer consisting of both same and opposite charged ions/molecules grows beyond the Stern layer; and, along with the Stern layer, the diffuse layer forms the EDL. Zeta potential spectroscopy helps in the determination of the colloidal stability and the surface charge on the NP. The composition of this diffuse layer is dynamic and varies depending on a variety of factors, e.g., pH, ionic strength, concentration, etc.

Liang et al. [56] conducted a study on the encapsulation of epigallocatechin gallate in zein/chitosan nanoparticles for controlled applications in food systems. They performed Zeta potential analysis using a laser light scattering instrument at 25 ℃ and a scattering angle of 90° and found that the particle size of EGCG-encapsulated zein/CS NPs was not as consistent as other formulations.

### 4.6. X-ray Photoelectron Spectroscopy (XPS)

X-ray photoelectron spectroscopy (XPS) is a surface-sensitive quantitative spectroscopic method that measures the elemental composition of the materials. X-ray photoelectron spectroscopy (XPS) is one of the most important and widely used surface analytical techniques. This technique provides chemical information about the surfaces. The principle of XPS is based on the generation of the X-ray by electron bombardment of a metal target. Mostly Al or Mg is used as the target source [57]. The energy from the resulting photons is directed towards the sample, which can eject the electrons. These incident X-rays are entirely absorbed by an atom. The injections of the electrons depend upon the amount of binding of the core electrons. If the binding energy is less than the photon energy, then the electrons may emit from the surface.

XPS can identify the elemental compositions of materials by measuring the Kinetic Energies (KEs) of their ejected electrons. XPS can detect the X-ray. XPS is sensitive to the chemical environments of the atoms it detects. The change in the chemical composition or a shift in the chemical composition is responsible for the change in the positions of the photoelectron signals, which can be helpful in identifying the chemical states of elements.

Barros et al. [58] used XPS analysis in their study to investigate the surface composition of NPs and the oxidation states of the Cu species to study the degradation of Amaranth food dye by heterogeneous electro-Fenton process. XPS analysis shows that there is a strong exclusion of copper species to the surface of the nanoparticle, as observed on copper spinel oxides.

## 5. Applications

### 5.1. Nanofood

Nanofood are those food products in which a nanoparticle or any nanostructured material is used at any stage of food development process (cultivation, production, processing and packaging). In other words, food developed by the addition of a nanomaterial or food that has been cultured, produced, processed or packaged using a nano-technological system is known as “nanofood” [59]. The concept of nanofood is developing day by day, as it has potential to fulfil essential requirements of food products; for example, it can enhance food safety, increase nutritional value, enhance flavour and reduce cost. Nanofood includes additives which help to achieve a longer shelf life, promote health or introduce a variety of new flavours. Antimicrobial agents packed in starch colloid are coated in such a way that if there is any microbial growth on the packed food, the antimicrobial agents will be released [60]. The presence of nano-sensors in food for the detection of pathogens makes the process quick, susceptible and effortless. Some food products also have nano-sized transport systems for the addition of nutrients and supplements, such as antioxidants, vitamins, minerals, flavours, colour and preservatives [59]. The nanocarrier system is added to protect the additives and ingredients during the processing of the food product. However, the Food and Drug Administration (FDA)issued a warning regarding nanofood products, as they have modifications in their physical and chemical properties. These nanofood products differ from the properties of the macroparticles of the same elements and may cause toxicity on interaction with a biological system [61]. Some examples of nanofoods are fortified fruit juices (High Vive, USA), NanoSlim beverage, tomato carotenoid in synthetic form, benzoic acid, ascorbic acid, nanoceutical slim shakes (assorted flavours, RBC Lifesciences, Irving, TX, USA), isoflavanones, omega 3 fatty acid, supplements such as vitamins A and E, coenzyme-Q10, lutein, ß-carotene, oat nutritional drinks (assorted flavours, Toddler Health, Los Angeles, CA, USA) and many more [59]. A listing of nano-related food and beverages are provided by the Nanotech Project in its Nanotechnology Consumer Products Inventory (Table 1).

### 5.2. Nanoencapsulated Probiotics

**Figure 2:**
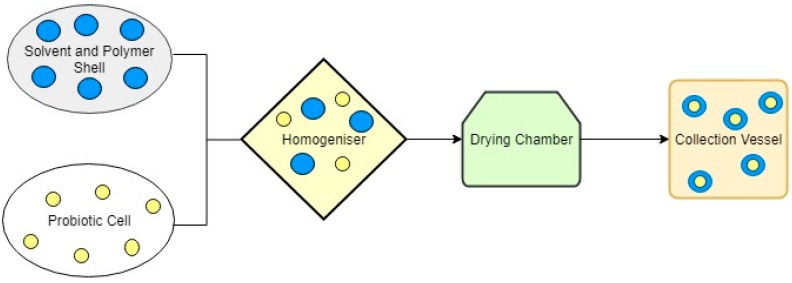
Schematic diagram of the probiotic encapsulation process.

Probiotics are the mixture of live bacterial species into yoghurts and yoghurt-type fermented milk, fruit-based drinks, cheese and pudding, which, when taken, have beneficial effect on the consumer. Its consumption balances the beneficial bacteria of our gut, improving gut health, enhancing the immune system of our body and lowering blood cholesterol level. The encapsulation of the product increases the shelf life of the food. Nanoencapsulation favours the development of specific bacterial probiotic, which can be delivered to the gastro-intestinal tract where it can interact with specific receptors [59]. In a study done by Vidhyalakshmi et al. in 2009, they used different encapsulated techniques encapsulating a health ingredient in order to achieve higher efficiency of the ingredient [62]. They also stated that the encapsulated probiotic material (Figure 2) had also improved viability with the acidic food product and survival strength in the harsh environmental conditions. Kalal et al. [63] encapsulated probiotic powder of bitter gourd juice and found that the viability of the *Lactobacillus casei* in the product is significantly good when encapsulated with maltodextrin.

### 5.3. Edible Nanocoatings

The development of nanotechnology in the food industry have led to the development of edible nano-coatings of about 5 nm thickness. Thin edible nano-coatings are created on the outer surface of fresh fruits and vegetables, raw meat, cheese, ready-to-eat foods and bakery goods. It acts as an obstruction to the exchange of gases and moisture that releases antioxidants, enzymes, flavour, colour and antimicrobial agents. This coating helps in the protection of food from spoilage and, hence, increases the shelf life of the food. Many edible coatings have been developed for fresh fruits and vegetables and raw meat. Dhital et al. developed an edible coating on “Chandler” strawberries to increase their shelf life [64]. Antibacterial edible nanocoating can be applied directly to bakery goods. Musso et al. [65] developed edible smart gelatin film based on curcumin and gelatin. The film has high antioxidant property and changes colour when it comes in contact with media at different pHs.

### 5.4. Nanocoatings for Food Contact Surfaces

Nanomaterial coating on food surfaces exhibits high-performance abilities for resisting the development of biofilm. Nanoparticles are used for the functionalisation and the controlled release of antimicrobial agents from surfaces, reducing the colonisation of the microorganisms, as well as inhibiting their propagation. These nanostructured materials are effective for a longer duration, prevent the growth of pathogenic bacteria and avoid food spoilage. Various nanoparticles, such as gold, silver, zinc oxide, titanium oxide and many more, have been studied as a coating for several food contact materials. Yemmireddy et al. developed a nanocoating of TiO_2_ for the surfaces of chopping boards and found significant microbial reduction on such surfaces [66]. Moreover, numerous products have been developed with the antimicrobial properties due to the presence of the nanoparticles that are available in the market (Table 2).

### 5.5. Active and Smart Packaging

Smart and active packaging systems, also referred as the intelligent packaging systems, are used to pack food and products in order to enhance desired attributes such as monitored transport and storage, increasing the shelf life of food, scrutinising freshness and releasing antimicrobial agents in order to reduce food contamination and spoilage. Bioengineered food packaging materials are closely related to intelligent, active and smart packaging [67]. Small sachets or packets of metals or their oxides, such as gold, silver, silica, aluminium oxide, zinc oxide, iron oxide, etc., are incorporated into the packaging. In many packaging systems, silver, nano zinc oxide and nano magnesium oxide have been used as antimicrobial agents. They have claimed to be active agents, as they inhibit microbial growth for a longer period of time. Nanostructure porous silicon food packaging enables the detection of pathogens and variations of temperature during food storage. Moreover, the development of next-generation packaging displays that include radio frequency identification display (RFID) as smart labels to assist in the quick and accurate distribution of foodstuffs with a limited shelf life.

Packaging can also be made smart by inducing a nano-sensor. Nano-sensors are tiny sensors attached either to food or food packaging. They can detect pathogenic bacteria, viruses and chemical contaminants in the food system [68]. They are highly efficient as one sensor can identify a wide range of pathogens. Nano-sensor research is focused on the rapid detection of contaminants in and around food and the tracking of food by the incorporation of a nano-sensor in the food packaging material.

Fand et al. [69] mentioned that packaging technology has a high impact on the quality and safety of meat. Intelligent packaging keeps a check on the condition of the product and exchanges information, while active packaging positively influences the internal environment of the packaging. Medina-Jaramillo et al. [70] developed a smart and active biodegradable film formed from natural extracts and starch. This film has high antioxidant properties, and its colour changes as there is a change in pH.

## 6. Conclusions

Food is a necessity of life, which provides us the energy to sustain life. The healthier the food, the more energy it will produce and maintain the metabolism. Nanotechnology works on the nanoscale level to introduce the nutritive components into food to make it healthier and safer. Nanoparticles could be incorporated in the food industry in any sector, such as processing, packaging, storage, transport, safety and quality improvement and security. Nano-nutritional supplements incorporated in food work with high efficacy. These particles are more easily accepted by cells of the body due to their size and surface morphologies. In contrast, there has always been a conflict between studies regarding the applications of nanotechnology in the food industry and the impact of nanoparticles on the health. Therefore, legislation has been developed on the use of nanotechnology in the food sector. Many nanofoods have been produced and commercialised in the market as a nutraceutical or food supplement. Nanotechnology is also used to enhance nutritive value, smart packaging, nano-sensing application and antimicrobial agents in the food system.

## Figures and Tables

**Table 1:** Products having nanocomposite materials.

Nanocomposite	Properties	Product
Silver	Food supplement, Antimicrobial promote cytotoxicity	Maternal Water (La Posta del Aguila), MesoSilver, Nano Silver dispersion, ASAP Health Max 30, Colloidal silver, Silver Biotics, Silver-22 TM
Gold	Food supplement	MesoGold^®^
Zinc oxide	Food supplement	MesoZinc, LifePak^®^ Nano
Titanium dioxide	Packaging materials, increased shelf life of food, antimicrobial	Hershey’s Chocolate Syrup, Albertson Chocolate Syrup, Cadbury Milk Chocolate Bar, parmesan cheese (Kraft Foods), M&M’s Chocolate Candy (Mars), Mentos Fresh Mint Gum, Nestlé Original Coffee Creamer, Best Food Mayonnaise, Lemon Lime Powerade (Coca-Cola), Oreo (Nabisco), Eclipse Spearmint Gum, Tic Tac Mints, Silk Original Soy Milk.
Lycopene	Nutraceutical	Synthetic lycopene
Silicon	Food and beverage supplement	MesoSilica ™ (Purest Colloids, Inc.), Microhydrin^®^ Products, Nanosiliceo Kapseln (Neosino), Nano-2 Bio-Sim
Calcium and magnesium	Stable and sustained release	24Hr Microactive^®^ CoQ10
Selenium	Antimicrobial protection	Nanotea (Shenzhen Become Industry & Trade Co., Ltd.)
Nanoemulsions	Antioxidant	Nanoemulsion (β-Carotene; α-Tocopherol) based ice-cream (Nestle, Unilever), Fabuless™
Micelles	Liquid carrier	Canola Active Oil (Shemen Industries), NovaSOL, Encapsome™, Aquanova^®^ Novasol^®^, Encapsome™
RBC’s NanoClusters	Delivery System	Nanoceuticals™ Slim Shake Chocolate
Bioregulators	Supplements	C.L.E.A.N. Products (SportMedix Inc.), B12 Rapid Shot™ (Priority one)

**Table 2:** Nanocomposite products for food contact surfaces.

Nanoparticle	Properties	Product
Silver	Antimicrobial protection	Oso fresh food storage container, Kinetic Go Green basic nanosilver food storage container, FresherLonger™ Miracle Food Storage, Fresher Longer^TM^ plastic storage bags, Primea Ring, Antibacterial Table Ware (Nano Care Technology, Ltd.), Daewoo^®^ Refrigerator, Nano Silver Cutting Board, Nurser
Ceramic	Hardness and strength	Bialetti^®^ Aeternum Saute Pan
Nanoclay	Hardness and strength, catalyst	Beer Bottle Plastics (Voridian), Nano flagon—Moon drunker, Durethan, Imperm (Nanocor)
Bentonite	Gas barrier	Nanoclay–polymer composites
Nanocomposites	Oxygen barrier	Aegis nylon 6 (Honeywell)
Luminesent protein	Microbial detection on surface	NanoBioluminescence (AgroMicron Ltd.)
Starch Nanosphere	Lower temperature of heat activation, adhesive containers	Ecosynthetix Adhesive
